# Structural studies of p53 inactivation by DNA-contact mutations and its rescue by suppressor mutations via alternative protein–DNA interactions

**DOI:** 10.1093/nar/gkt630

**Published:** 2013-07-17

**Authors:** Amir Eldar, Haim Rozenberg, Yael Diskin-Posner, Remo Rohs, Zippora Shakked

**Affiliations:** ^1^Department of Structural Biology, Weizmann Institute of Science, Rehovot 76100, Israel and ^2^Molecular and Computational Biology Program, University of Southern California, Los Angeles, CA 90089, USA

## Abstract

A p53 hot-spot mutation found frequently in human cancer is the replacement of R273 by histidine or cysteine residues resulting in p53 loss of function as a tumor suppressor. These mutants can be reactivated by the incorporation of second-site suppressor mutations. Here, we present high-resolution crystal structures of the p53 core domains of the cancer-related proteins, the rescued proteins and their complexes with DNA. The structures show that inactivation of p53 results from the incapacity of the mutated residues to form stabilizing interactions with the DNA backbone, and that reactivation is achieved through alternative interactions formed by the suppressor mutations. Detailed structural and computational analysis demonstrates that the rescued p53 complexes are not fully restored in terms of DNA structure and its interface with p53. Contrary to our previously studied wild-type (wt) p53-DNA complexes showing non-canonical Hoogsteen A/T base pairs of the DNA helix that lead to local minor-groove narrowing and enhanced electrostatic interactions with p53, the current structures display Watson–Crick base pairs associated with direct or water-mediated hydrogen bonds with p53 at the minor groove. These findings highlight the pivotal role played by R273 residues in supporting the unique geometry of the DNA target and its sequence-specific complex with p53.

## INTRODUCTION

The tumor suppressor p53 acts as a DNA sequence-specific transcription factor regulating and activating the expression of a range of target genes in response to genotoxic stress. This in turn initiates a cascade of signal transduction pathways leading to different cellular responses including cell-cycle arrest and apoptosis that are known to prevent cancer development (1–4). p53 binds as a tetramer to specific response elements consisting mainly of two decameric half-sites separated by a variable number of base pairs (5–7). Mutations in the p53 gene that lead to inactivation of the protein are observed in ∼50% of human cancers (8,9). The majority of tumor-related p53 mutations, particularly those defined as mutational ‘hotspots’, occur within the DNA-binding core domain of p53 (10). The top hotspot mutations are located at or near the protein–DNA interface and can be divided into two major groups: DNA-contact mutations affecting residues involved directly in DNA contacts without altering p53 conformation, and structural mutations that cause a conformational change in the core domain (11). R273, a DNA-contact amino acid, is one of the most frequently altered residues in human cancer (6.4% of all somatic mutations), with mutations to histidine (46.6%) and to cysteine (39.1%) being most common (8,9).

Crystal structures of the p53 core-domain bound to DNA (12–17) show that the positively charged guanidinium groups of R273 residues interact with the negatively charged DNA backbone at the center of each DNA half-site, supported by salt-bridge and hydrogen-bond interactions. As discussed previously, R273 residues play a pivotal role in docking p53 to the DNA backbone at the central region of each half-site where no direct base-mediated contacts exist (13). Substitution of R273 by histidine or cysteine, referred to as R273H and R273C, leads to dramatic reduction in the DNA binding affinity, even though the protein retains wild-type stability (18).

The reactivation of mutant p53 by various pathways faces a common challenge: reversing the effect of a single amino acid mutation in the core domain, thus restoring its natural function. It has been shown by DNA binding, transcriptional activation and tumor-suppressing assays that the incorporation of a second mutation into oncogenic p53, referred to as second-site suppressor mutation, can rescue the normal activity of p53 as described later in the text for the current hot-spot mutations. In the case of R273H and R273C, it was shown that the replacement of threonine by arginine at position 284 (T284R) restores activity to both p53 mutants (19). Replacing serine by arginine at position 240 (S240R) was also found to rescue R273H (20). Although S240R alone was found to act as a suppressor mutation only for R273H, it was shown that in combination with either T123A or H178Y, S240R can suppress the effect of R273C mutation on p53 function (20). These observations and the fact that both R273H and R273C mutations have similar effects on p53 structure and function (21) suggest that S240R alone might also rescue R273C. In a more recent screen of p53 second-site suppressor mutations, R273H was also shown to be rescued by H178Y (22).

To elucidate the structural basis of p53 dysfunction as a result of DNA-contact mutations and the mechanisms of their rescue by second-site suppressor mutations, we crystallized and analyzed the structures of the corresponding single and double p53 mutants using the core domain of wild-type human p53 as a template. These include the oncogenic mutants R273H and R273C, the rescued proteins harboring each of the aforementioned mutations together with a second-site suppressor mutation, T284R or S240R, and their sequence-specific complexes with consensus DNA-binding sites. The comparative analysis of the various structures shows that inactivation of the cancer-related mutants results from the lack of stabilizing interactions between p53 and the DNA target. Reactivation of these proteins is achieved by new interactions formed by the second-site mutations, T284R or S240R, with the DNA backbone. However, the protein–DNA interface at the center of each DNA half-site is distinctly different from that of the wt complex in terms of DNA shape and minor-groove interactions.

## MATERIALS AND METHODS

### Proteins and DNA

A pET-27 b plasmid (Novagen) carrying the sequence that encodes the core domain of human wild-type p53 (corresponding to residues 94–293) was used as a template in a Quickchange® site-directed mutagenesis reaction (Stratagene/Agilent Technology) as previously described (23). The resulting plasmids contained a sequence corresponding to the core domain that incorporates the single mutation R273C or R273H. These plasmids were used as templates for a second mutagenesis reaction to obtain plasmids corresponding to the following double mutants: R273H/T284R, R273H/S240R, R273C/T284R and R273C/S240R. *Escherichia coli* BL21 (DE3) cells (Novagen) were transformed with the plasmids, and the expressed proteins were purified by ion exchange followed by a gel-filtration chromatography, pursuing a procedure described previously (13,23). The proteins were concentrated to 3.5–8.5 mg/ml depending on their solubility, aliquoted and stored at −80°C.

A self-complementary DNA oligonucleotide carrying p53 DNA half-site of the sequence 5′-cGGGCATGCCCg-3′ (consensus sequence underlined) was purchased after standard desalting and lyophilization from IDT (Integrated DNA Technologies, Israel) and purified by ion-exchange chromatography. The DNA was then dialyzed against water, lyophilized and dissolved in water to obtain a concentration of 20 mg/ml.

### Crystallization and data collection and processing

Crystals of the proteins and protein–DNA complexes with DNA were grown at 19°C by the hanging-drop vapor-diffusion method (24) from 4 μl of drops equilibrated against a 0.5 ml of reservoir solution. Initial crystallization experiments were performed using the Hampton Research PEG/Ion screen. Crystallization conditions were optimized using homemade solutions. Final crystallization conditions are given in Supplementary Table S1. Before data collection, crystals were transferred into a cryo-protectant for few minutes. Paratone-N oil (Hampton Research) was successfully used for most of the crystals. When needed, alternative cryo-stabilization solutions were prepared mimicking the mother liquor supplemented with glycerol or ethylene glycol at concentrations leading to efficient cryo-protection. The protected crystals were mounted on Hampton Research CryoCapHT nylon loops and flash-cooled in a stream of nitrogen gas at 100 K using an Oxford Cryostream 700-series for in-house measurements or plunged and stored into liquid nitrogen for further measurements.

Diffraction data were collected at the Weizmann Institute X-ray Crystallography Laboratory on Rigaku R-AXIS IV++ Imaging Plate detector mounted on Rigaku RU-H3R generator with CuKα radiation (1.5418 Å) focused by Osmic confocal mirrors, and at the European Synchrotron Radiation Facility (ESRF, Grenoble, FRANCE) at beam lines ID14-2, ID14-3, ID23-1 and ID23-2 (see Tables 1 and 2 for details).

**Table 1.** Data collection statistics of R273H-related structures

Data sets	R273H (form I)	R273H (form II)	R273H/T284R	R273H/T284R-DNA^a^	R273H/S240R
PDB ID	4IBS	4IJT	4IBT	4IBW	4IBY
X-Ray source					
Beamline	RU-H3R	ESRF ID14-3	RU-H3R	ESRF ID14-2	ESRF ID14-3
Wavelength (Å)	1.54178	0.931	1.54178	0.933	0.931
Detector	R-AXIS-IV^++^	ADSC Q4R	R-AXIS-IV^++^	ADSC Q4	ADSC Q4R
Diffraction data					
Space group	*P*2_1_	*P*6_1_	*P*2_1_	*C*2	*P*2_1_
Cell dimensions:					
a,b,c (Å)	68.9,70.2,83.7	74.9,74.9,73.3	68.9,70.2,84.0	137.6,49.9,34.2	43.5,69.2,67.2
α,β,γ (°)	90,90.0,90	90,90,120	90,90.0,90	90,93.7,90	90,96.6,90
No. of proteins/DNA duplexes in a.u.	4	1	4	1/0.5	2
Resolution (Å)	32-1.78	27-1.78	36-1.7	40-1.79	25-1.45
Upper resolution shell (Å)	1.81-1.78	1.81-1.78	1.73-1.70	1.82-1.79	1.47-1.45
Measured reflections	541 703	112 980	693 820	161 770	290 925
Unique reflections	74 257(3178)^b^	22 336(1116)	82 678(3910)	21 929(1072)	69 956(3472)
Completeness (%)	97.1(83.1)	99.7(100)	93.6(89.3)	100(100)	99.7(100)
Average I/σ(I)	33.1(4.1)	12.1(3.1)	37.7(3.6)	16.7(3.6)	18.1(2.6)
R_sym_ (I) (%)	5.9(33.5)	14.1(52.9)	5.4(53.7)	11.7(53.9)	7.7(53.7)
Twin law/twin fraction (%)	h,-k,-l/36.0		h,-k,-l/15.0		

^a^The DNA sequence is cGGGCATGCCCg, consensus sequence underlined.

^b^The values in parentheses refer to the data of the corresponding upper resolution shells.

**Table 2.** Data collection statistics of R273C-related structures

Data sets	R273C	R273C/T284R	R273C/T284R-DNA^a^	R273C/S240R-DNA^a^
PDB ID	4IBQ	4IBZ	4IBU	4IBV
X-Ray source				
Beamline	ESRF ID14-3	ESRF ID23-1	ESRF ID14-2	ESRF ID23-2
Wavelength (Å)	0.931	0.9759	0.933	0.8726
Detector	ADSC Q4R	MarMosaic225	ADSC Q4	MarMosaic225
Diffraction data				
Space group	*P*2_1_	*P*2_1_	*P*1	*C*2
Cell dimensions:				
a,b,c (Å)	68.7,69.7,83.7	68.7,70.4,84.6	54.5,58.2,78.0	137.6,50.3,34.0
α,β,γ (°)	90,90.1,90	90,89.9,90	83.0,87.8,73.6	90,93.5,90
No. of proteins/DNA duplexes in a.u.	4	4	4/2	1/0.5
Resolution (Å)	30-1.8	27-1.92	40-1.69	50-2.1
Upper resolution shell (Å)	1.83-1.80	1.95-1.92	1.72-1.69	2.14-2.10
Measured reflections	467102	220453	376963	82645
Unique reflections	71 943(3554)^b^	60 012(2501)	96 978(3280)	13 264(556)
Completeness (%)	97.9(96.9)	97.8(81.2)	94.7(64.4)	96.6(83.6)
Average I/σ(I)	27.9(5.4)	14.1(2.2)	26.7(3.3)	21.70(4.2)
R_sym_ (I) (%)	6.7(32.8)	8.1(35.6)	4.7(35.5)	8.2(25.5)
Twin law/twin fraction (%)	h,-k,-l/48.0	h,-k,-l/7.0		

^a^The DNA sequence is cGGGCATGCCCg, consensus sequence underlined.

^b^The values in parentheses refer to the data of the corresponding upper resolution shells.

On exposure to X-ray radiation, no significant decay was observed in the diffraction intensities and hence in each case, a complete data set could be obtained from a single crystal. The program BEST (25) was used to optimize the X-ray data collection strategy, in particular, for the high-resolution data. The data were indexed and integrated with DENZO and scaled with SCALEPACK as implemented in HKL-2000 (26). Data collection statistics is given in Tables 1 and 2.

### Structure determination and refinement

Before structure determination, each data set was subjected to lattice and twinning analyses using Xtriage from the Phenix package (27). Four data sets from the isomorphic monoclinic crystals with β≈90° were shown to be affected by pseudo-merohedral twinning. The twin target function was used in the refinement of these structures (see details in Tables 1 and 2).

Most of the new crystal structures are isomorphic to previously published structures of the p53 core domain (28) and its complexes with DNA (13,16,23). To minimize bias and errors, the new structures were solved independently by molecular replacement, using Molrep or AMoRe from the CCP4 package (29) or Phaser (30) as implemented in the Phenix package (27). The crystal structure of the human p53 core domain (chain A of PDB ID 2AC0 or 1TSR) was used as a search model. To minimize bias toward the starting model, each refinement was initiated with a slow-cooling simulated annealing cycle using Phenix (27). Successive rounds of model building and manual corrections with COOT (31) and refinement with Phenix (27) were performed to build the complete models.

The DNA molecules in the protein–DNA complexes were built based on the corresponding SigmaA and SigmaD electron density maps. For each crystal structure, an R-free data set was used and kept throughout the process to monitor the refinement progress. Refinement statistics is summarized in Tables 3 and 4.

**Table 3.** Refinement statistics of R273H-related structures

Data sets	R273H (form I)	R273H (form II)	R273H/T284R	R273H/T284R-DNA^a^	R273H/S240R
Refinement					
Resolution range (Å)	25.1-1.78	24.5-1.78	35.9-1.70	34.3-1.79	21.8-1.45
No. of reflections (I/σ(I) > 0)	74239	22322	82662	21923	69847
No. of reflections in test set	2564	1107	2599	1114	3524
R-working (%)/R-free (%)	16.3/18.1 ^b^	16.2/19.4	17.0/18.3 ^b^	14.2/18.8	16.9/20.0
No. of protein/DNA atoms	6333	1669	6477	1653/225	3308
No. of Zn atoms	4	2	4	1	2
No. of solvent atoms	653	292	971	425	808
Overall average B factor (Å^b^)	21	17.4	23.5	16.1	14.5
Root mean square deviations:					
Bond length (Å)	0.004	0.011	0.004	0.006	0.007
Bond angle (°)	0.88	1.33	0.86	1.15	1.13
Ramachandran plot^c^					
Most favored (%)	98.9	100	98.5	99.5	99
Additionally allowed (%)	1.1	0	1.5	0.5	1
Disallowed (%)	0	0	0	0	0

^a^The DNA sequence is cGGGCATGCCCg, consensus sequence underlined.

^b^Refinement target: TWIN_LSQ_F.

^c^Derived by *PROCHECK* (32).

**Table 4.** Refinement statistics of R273C-related structures

Data sets	R273C	R273C/T284R	R273C/T284R-DNA^a^	R273C/S240R-DNA^a^
Refinement				
Resolution range (Å)	29.1-1.8	26.7-1.9	30.1-1.7	47.2-2.1
No. of reflections (I/σ(I) > 0)	71927	59996	95621	13.262
No. of reflections in test set	3686	2544	4783	667
R-working (%)/R-free (%)	13.4/16.3 ^b^	17.9/23.3 ^b^	16.3/19.7	16.0/23.1
No. of protein/DNA atoms	6089	6205	6422/975	1609/221
No. of Zn atoms	4	4	4	1
No. of solvent atoms	944	720	1058	211
Overall average B factor (Å^b^)	19.3	33.1	26.8	28.0
Root mean square deviations:				
Bond length (Å)	0.006	0.007	0.006	0.007
Bond angle (°)	0.99	1.07	1.17	1.22
Ramachandran plot^c^				
Most favored (%)	98.5	97.9	98.7	98.5
Additionally allowed (%)	1.4	2.1	0.7	1
Disallowed (%)	0.1	0	0.6	0.5

^a^The DNA sequence is cGGGCATGCCCg, consensus sequence underlined.

^b^Refinement target: TWIN_LSQ_F.

^c^Derived by *PROCHECK* (32).

### DNA structure analysis and electrostatic potential calculation

DNA structural features (helix diameter and minor-groove width) were calculated with Curves 5.3 (33) modified to incorporate Hoogsteen base pairs as described previously (16). Electrostatic potential as a function of base sequence was calculated with DelPhi (34) at physiologic ionic strength 0.145 M using a previously described protocol (16,35).

## RESULTS AND DISCUSSION

### Global and local structures of the core-domain mutants and their complexes with DNA

**Figure 1.**
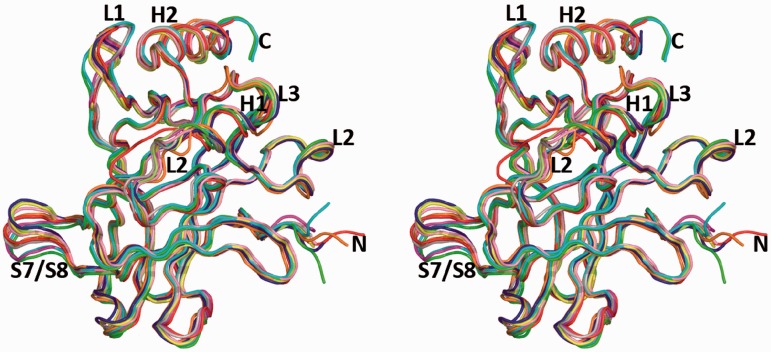
Stereo view of the R273-related mutants superposed on the wt core domain. The color code is wt (PDB ID 2AC0 molecule C) in gray, R273H (form I) in yellow, R273H (form II) in red, R273C in lime, R273H/S240R in orange, R273C/T284R in pink, R273H/T284R in blue, R273C/S240R-DNA in green, R273H/T284R-DNA in cyan and R273C/T284R-DNA in magenta (the color code is maintained throughout the figures).

The crystal structures provide high-resolution information on various R273-related mutants including the oncogenic single mutants: R273H and R273C, the rescued double mutants: R273H/T284R, R273C/T284R, R273H/S240R and R273C/S240R, and their complexes with DNA. The secondary structures of the various mutants are similar to that of the wt protein (Supplementary Figure S1). A superposition of the mutant structures onto one of the wt core domain structures shows that their 3D structures are similar to that of the wt protein, demonstrating that mutations located at the protein surface have a negligible effect on the structure of the protein (Figure 1). These findings are in agreement with the crystal structure data of R273H and R273C mutants analyzed in the context of the thermostable core domain, which incorporates four stabilizing mutations (21,36).

**Figure 2.**
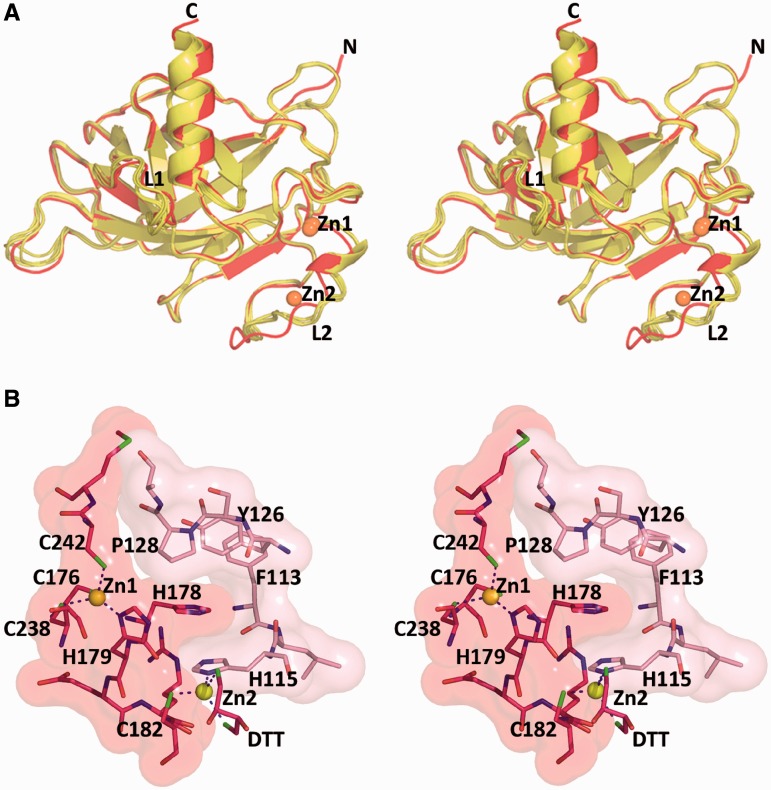
Comparison of the two structural variants of R273H. (**A**) Stereo view of the structure of R273H (form II) (red) superposed on the structure of R273H (form I) (yellow), showing the conformational changes in the L2 region. Also shown are the primary zinc atoms (Zn1), and the second zinc atom (Zn2) in R273H (form II). This view is different than that of Figure 1 to highlight the changes in L2 and the location of the different Zn atoms. (**B**) Stereo view of the intermolecular interface formed by symmetry-related molecules in R273H (form II). Zn1 is the physiological zinc atom common to all p53 structures, bound to C176, H179, C238 and C242. Zn2 is the second ion bound to C182 of one molecule (red), to H115 from a symmetry-related molecule (pink) and to the thiol groups of a DTT molecule (red).

Significant variability, however, is observed in flexible regions such as the DNA-binding loop L1, which exhibits different conformations between the free and DNA-bound p53 as shown previously (12–17). A new conformational variant is observed here in the L2 loop of one of the two R273H crystal structures, referred to as R273H (form II) in Tables 1 and 3. The major changes are shown by the backbone conformation of residues 182–187 next to the H1 helix (Figure 2A). In addition to the primary Zn atom (Zn1), which is common to all core-domain structures, this structural variant contains a second Zn atom (Zn2), linking two adjacent molecules related by crystal symmetry. Whereas the first Zn atom is of functional importance, as it supports the core-domain integrity and dimerization on binding to DNA (13), the second Zn atom observed in R273H (form II) is coordinated to C182 of one molecule, H115 of the symmetry-related molecule and the thiol groups of a trapped DTT (dithiothreitol) molecule (Figure 2B). The interface between the two molecules is further stabilized by the side chain of H178 from one molecule positioned in a pocket created by three amino acids (H115, Y126 and P128) from the second molecule (shown in Figure 2B) and hence interferes with DNA binding. In this manner, a continuous chain of p53 molecules linked by zinc atoms is formed in the crystal via the crystallographic 6_1_ axis (Supplementary Figure S2).

**Figure 3.**
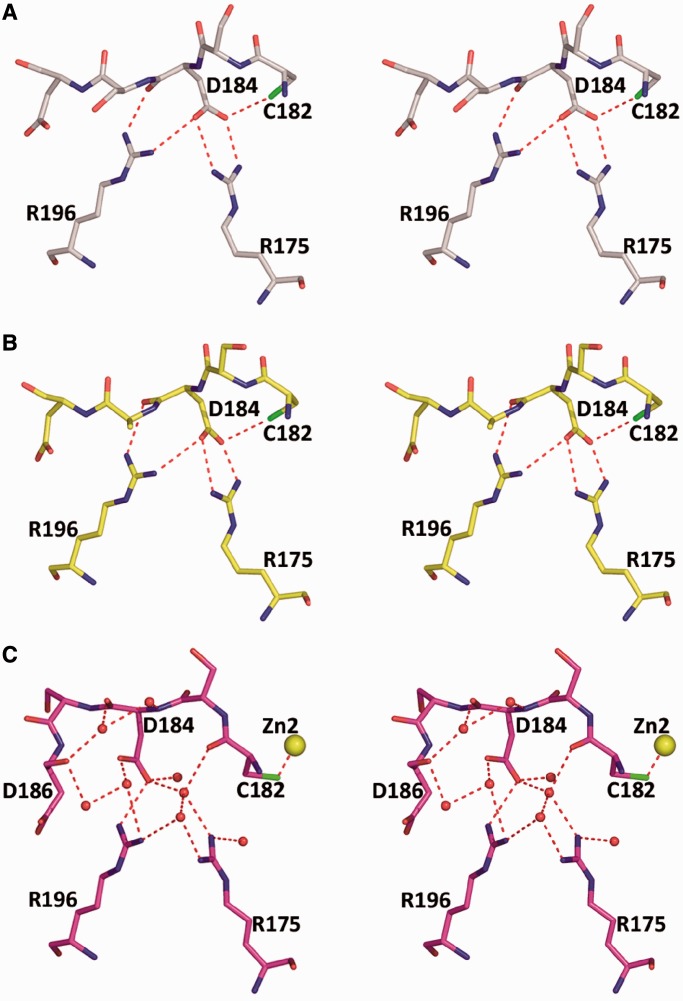
Interactions between the L2 loop and R175. (**A**) wt core-domain structure bound to DNA (PDB ID 2AC0). R175 forms a bidentate salt bridge with D184 supported by hydrogen bonds with C182 and R196. (**B**) The structure of the same region in R273H (form I) is similar to that of the wt protein. (**C**) The structure of the same region in R273H (form II) showing alternative supporting interactions between R175 and L2 loop formed by water-mediated hydrogen-bonded network. The second zinc is shown (yellow sphere).

Furthermore, the conformational changes in L2 as a result of the non-physiological Zn coordination disrupt the salt bridges between R175 and D184 that have been shown by most structures including R273H (form I) and by the previously reported structures of the wild-type (wt) core domain (13,28). These salt-bridge interactions (shown in Figure 3A and B) are supported by the side chains of C182 and R196. R175 is one of the top six hot-spot residues, which is most frequently mutated to histidine in human cancer. The loss of the aforementioned interactions leading to destabilization of the core domain appears to contribute to the deleterious effect of this mutation as described later in the text. In the conformational variant displayed by R273H (form II), R175 is engaged in water-mediated hydrogen bonds with C182, D184 and R196 (Figure 3C). The interruption of salt bridges via R175 (loss of enthalpy) and the incorporation of water molecules (loss of entropy) indicate that the core domain structure of form II is destabilized relative to that of form I. It has been shown previously that although Zn is essential for the structural integrity of p53, slight stoichiometric excess of free Zn^2+^ traps the p53 core domain in a misfolded state in which Zn^2+^ is bound to non-physiological ligands (37,38). The zinc-mediated interactions between core-domain molecules observed here indicate that binding of another Zn^2+^ to p53 via cysteine and histidine residues could facilitate p53 destabilization and aggregation, and hence loss of function.

**Figure 4.**
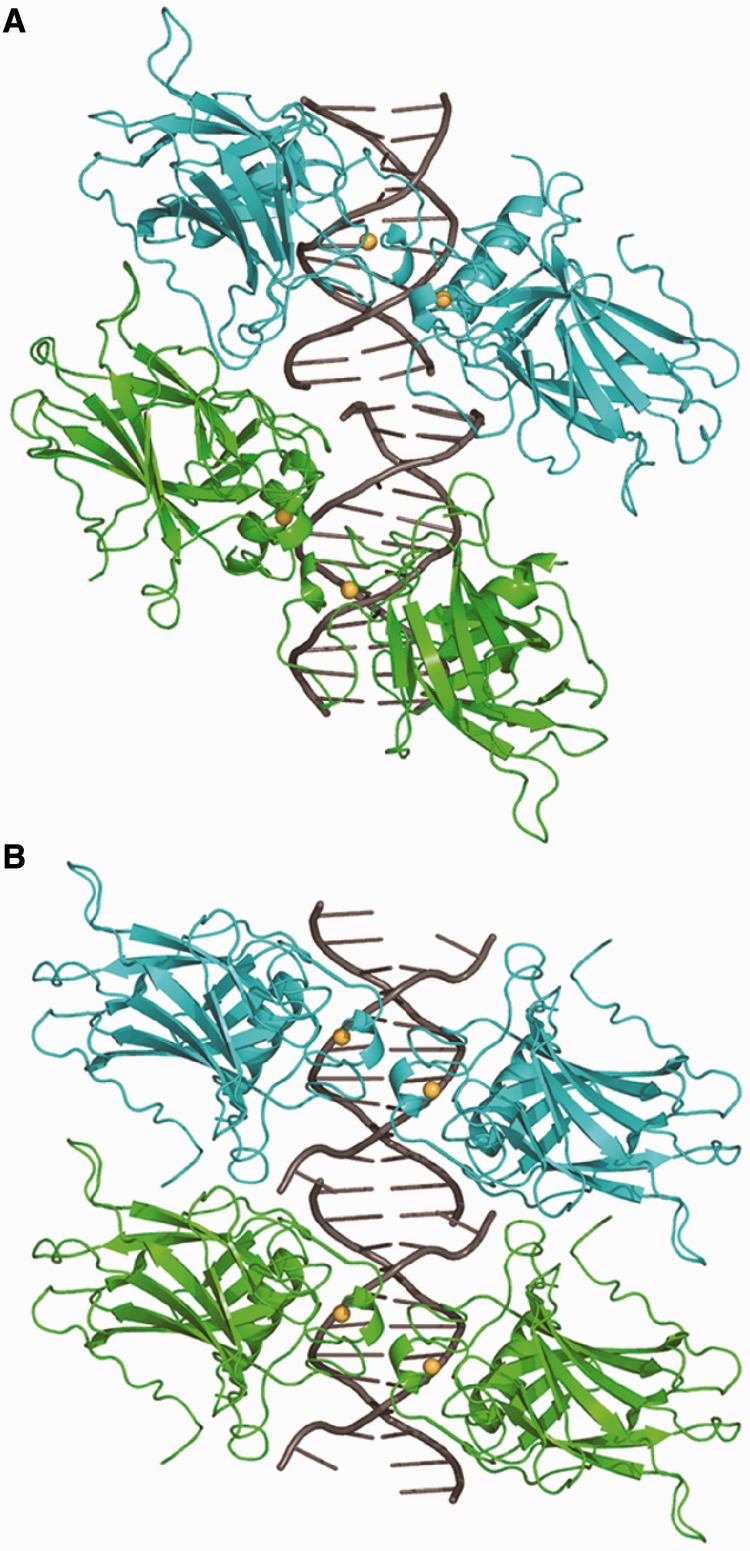
Type I and type II complexes of the rescued proteins. (**A**) Type I complex shown by R273C/T284R-DNA. Here, the two DNA half sites (gray) are separated by two base pairs, and the two dimers (shown in cyan and green) are rotated relative to each other. (**B**) Type II complex is shown by both R273H/T284R-DNA and R273C/S240R-DNA structures. The figure is based on the structure of the former complex. Here, the two half sites are contiguous, and the two dimers are parallel to each other.

Crystal structures of p53 rescued proteins in complexes with DNA were obtained for three double mutants: R273H/T284R, R273C/T284R and R273C/S240R. Similarly to the structures of the wt core domain and the rescued mutant R249S/H168R bound to DNA studied previously by our group (13,16,23), two types of tetrameric complexes (type I and type II) were obtained with the same DNA dodecamer incorporating a consensus half-site (cGGGCATGCCCg), shown in Figure 4. In the first case, two dodecameric duplexes bound to two p53 core-domain dimers are stacked end-to-end, and hence the decameric half-sites are separated by two base pairs. In the second case, two bases at the 3′-end of each strand (C-G) are extra-helical and the 5′-terminal C base of a symmetry-related strand completes the consensus half-site to form a contiguous 20-bp binding site (illustrated in Supplementary Figure S3). We have previously demonstrated that this binding site is highly similar, in terms of DNA geometry and protein–DNA interface, to that of a covalently linked 20 bp DNA (16).

### Structural basis of p53 loss of function and its rescue by second-site suppressor mutations

**Figure 5.**
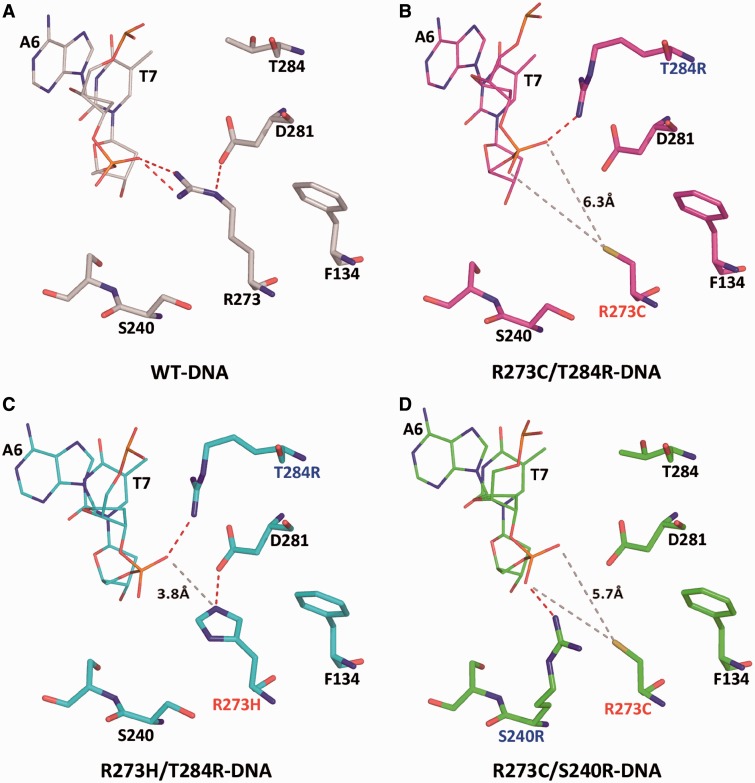
Close-up views of the mutation sites in the rescued p53 proteins bound to DNA, compared with the wt p53-DNA complex. (**A**) Wild-type p53-DNA interface showing the interaction site of R273 (PDB ID 2AC0). (**B–D**) The corresponding sites of the rescued proteins, indicating that both R273H and R273C side chains are too short to interact with the DNA backbone. The corresponding distances to the DNA are shown by gray dashed lines. Alternative hydrogen bonds to the DNA backbone are formed by the second-site suppressor mutations, T284R and S240R, shown by red dashed lines. Black labels denote wt residues. Red and blue labels denote primary and suppressor mutations, respectively.

As described previously, R273 side chains from four p53 molecules interact directly with four symmetrically disposed phosphate groups of the DNA backbone at the center of each DNA half-site, thereby stabilizing the tetrameric p53-DNA complex (13). As illustrated in Figure 5A, the guanidinum group of R273 makes two hydrogen bonds with a non-esterified oxygen atom of the DNA backbone. The unique conformation of the R273 side chain that facilitates this interaction is buttressed by an aspartate group from D281.

Comparison between the wt core domain and the various R273-related mutants demonstrates that loss of function brought about the two DNA-contact mutations, R273H or R273C, is caused by the incapacity of the shorter side chains of these amino acids to form stabilizing interactions with the DNA. In the reactivated double mutants bound to DNA, the shortest distances of the mutated amino acids, histidine and cysteine, from the DNA backbone are close to 4 and 6 Å, respectively (Figure 5B–D). Similar geometrical features of the protein surfaces are displayed by the crystal structures of the single mutants: R273H and R273C, and of the double mutants: R273H/S240R, R273H/T284 and R273C/T284R in the absence of DNA. The mutation sites of these proteins are compared with the corresponding region of the wt p53-DNA complex by modeling the central A/T doublet as shown in Supplementary Figure S4, confirming again the inability of His or Cys residues at position 273 to interact with the DNA target. The arginine side chains of the second-site mutations in these structures (T284R and S240R) display a range of conformations in their free state (Supplementary Figure S4), but a single conformation is observed in their DNA-bound state (Figure 5B–D).

The protein–DNA interfaces of the three crystal structures reveal the rescue mechanism of each of the suppressor mutations T284R and S240R. The loss of protein–DNA contacts as a result of the primary mutations, R273H or R273C, is compensated for by a new interaction between the side chain of T284R with the same DNA phosphate oxygen shown to interact with R273 in the wt p53-DNA complex (Figure 5A–C). A similar rescue mechanism is displayed by the second suppressor mutation, S240R, where the arginine side chain approaches the DNA backbone from the opposite direction interacting with the second non-esterified oxygen atom of the same phosphate group (Figure 5D). However, as described later in the text, the protein–DNA interface at the center of each DNA half-site is not restored in terms of DNA shape and minor-groove interactions.

### DNA shape and protein–DNA interactions

The overall architectures of the rescued p53-DNA complexes are similar to their wt counterparts for both type I and type II tetrameric complexes. The crystal structure of R273C/T284R-DNA (space group *P*1) is similar to that of type I complexes with a two base-pair spacer between the DNA half-sites (13,23) (shown in Figure 4A). The crystal structures of R273H/T284R-DNA and R273C/S240R-DNA (space group *C*2) are similar to that of type II complexes with contiguous half-sites (16) (shown in Figure 4B). However, a detailed comparison of the DNA conformation and protein–DNA interfaces of these structures reveals significant differences between the wt and the rescued complexes. In particular, major alterations are observed in type II complexes at the central A/T base pairs of the DNA half-sites.

**Figure 6.**
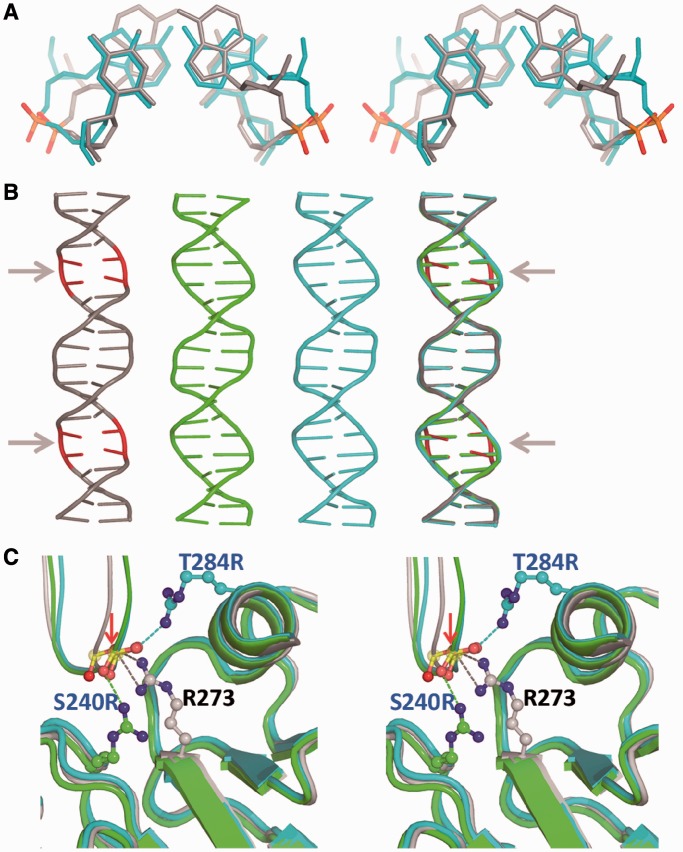
The effect of base-pairing geometry on DNA shape in type II complexes. (**A**) Stereo view of the A/T dinucleotide pairs at the center of the DNA half-site, showing the local backbone shift and minor-groove narrowing caused by Hoogsteen base pairs in the wt complex (gray, PDB ID 3IGL) relative to Watson–Crick base pairs of the rescued complex R273H/T284R-DNA (cyan). (**B**) Comparison of three DNA helices bound to the wt and rescued p53. The color code is gray for wt-DNA (PDB ID 3IGL), cyan for R273H/T284R-DNA and green for R273C/S240R-DNA. Also shown is a superposition of the three helices. The continuous 20 bp long helices were obtained by modeling the missing phosphate groups in the full-length binding site (see scheme **B** in Supplementary Figure S3). The overall shape of the three DNA helices is similar except for the constriction shown by the wt DNA at the center of each half site, caused by the Hoogsteen base-pairing geometry (highlighted in red and indicated by arrows). (**C**) Close-up stereo view of the interaction modes of R273, T284R and S240R with their DNA-binding sites, showing the shift in the DNA backbone of the wt complex (gray) relative to those of the rescued complexes (cyan and green). The red arrow points to the position of the oxygen atom interacting with R273. Only one interaction site is shown for each complex. The other three sites are equivalent by symmetry.

Contrary to the non-canonical Hoogsteen base-pair geometry displayed by the A/T base pairs in type II complexes of wt p53 (16), the Watson–Crick geometry is observed in the current structures as illustrated in Figure 6A and Supplementary Figure S5. It should be emphasized that the observed change in base-pairing geometry is not driven by crystal packing interactions because the new crystal structures are isomorphous to the wt crystal structure, and the DNA targets are identical. We have previously shown that the Hoogsteen geometry of the A/T doublet leads to significant local compression of the helix diameter at the center of each half-site associated with distinct minor-groove narrowing at regions flanking the CATG tetranucleotides (16). The effect of the different base-pair geometries on the local shape of the DNA helix in these regions is shown in Figure 6A and B. To understand the structural origin of the drastic geometrical change in the base-pairing configuration, we compared the protein–DNA interfaces at the interaction sites of R273 and of the two rescuing mutations, T284R and S240R as shown in Figure 6C. In the wt complex, the two amino groups of R273 are oriented toward the central backbone of the DNA half-site forming two hydrogen bonds with the phosphate group. In the two rescued complexes, the arginine side chains of the suppressor mutations, T284R and S240R, protrude from opposite directions toward the DNA, each forming a single interaction with the same phosphate group, but with different non-esterified oxygen atoms (Figure 6C). The directed interactions of the R273 amino acids with the DNA backbone appear to support the local constriction of the double helix prompted by the two Hoogsteen base pairs, whereas the weaker interactions via T284R or S240R appear incapable of supporting this change, thus retaining a continuously regular double helix with Watson–Crick base pairs. As illustrated in Figure 6C, the DNA strand interacting with R273 (shown in gray) is shifted toward the helix center relative to the equivalent strands interacting with T284R and S240R (shown in cyan and green, respectively).

**Figure 7.**
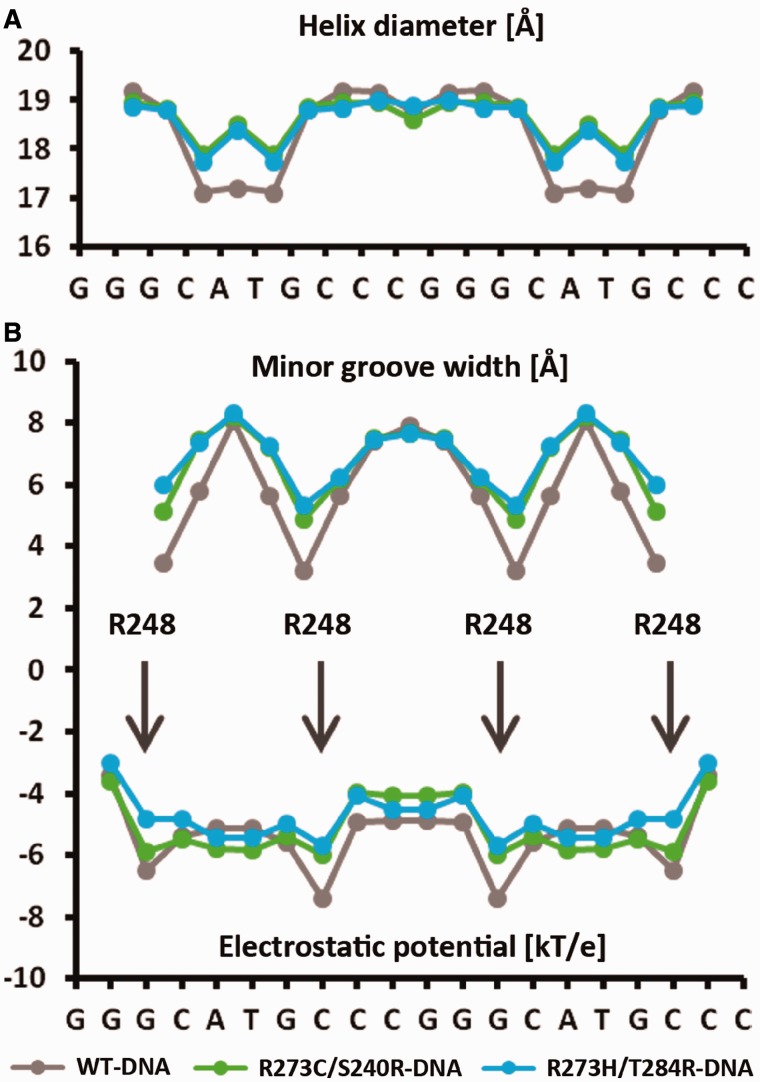
DNA helix parameters and electrostatic potential in type II complexes. (**A**) Helix diameter as a function of the base sequence showing the compression of the DNA helix at the center of each half-site. A larger effect is displayed by the DNA helix of the wt complex (shown in gray) as a result of the Hoogsteen geometry of the A/T base pairs at each half-site. (**B**) Minor-groove width and the corresponding electrostatic potential as a function of the base sequence. The four minor-groove minima are aligned with the electrostatic potential minima. The positions of the four R248 residues are indicated by arrows.

The variations in the DNA helix diameter and minor-groove width of the wt complex and those of the current crystal structures are shown in Figure 7. We have previously shown that the distinct narrowing of the minor groove as a result of the Hoogsteen geometry leads to enhanced negative electrostatic potential at the four regions close to the interaction sites of the positively charged R248 residues. Such electrostatic interactions at the minor groove contribute to the stabilization of the wt p53-DNA complex, highlighting the role of DNA shape in its recognition by p53. In the current structures, however, as a result of the Watson–Crick geometry of the A/T base pairs, the corresponding minor-groove regions are shallower, and their associated electrostatic potential is less negative, resulting in a relatively smaller contribution to complex stabilization through electrostatic interactions (Figure 7B).

**Figure 8.**
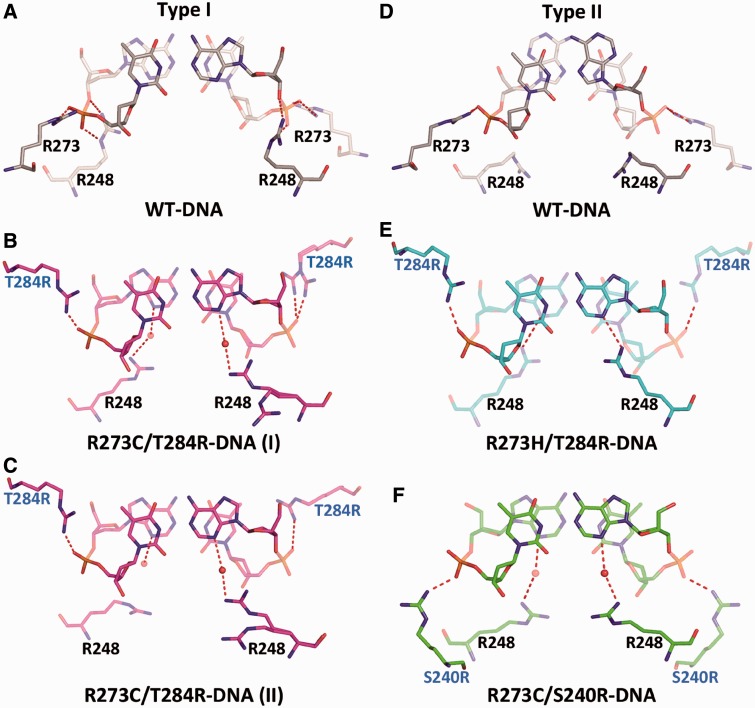
The A/T dinucleotide pairs at the center of the DNA half-site and their interactions with the wt and rescued p53. (**A**) In type I complex of wt-DNA (PDB ID 2AC0), the A/T base pairs display the Watson–Crick geometry. R273 side chains interact with the DNA phosphate groups (also shown in Figure 5A). The R248 residues interact occasionally with the backbone phosphates (shown here) or via water molecules (not shown). (**B** and **C**) In type I complex of R273C/T284R-DNA, the two dinucleotide pairs display the Watson–Crick geometry. T284R side chains interact with the backbone phosphate (also shown in Figure 5B). The R248 side chains at the minor groove are occasionally disordered and form water-mediated hydrogen bonds with the N3 atoms of the adenine bases. (**D**) In type II complex of wt-DNA, the A/T base pairs display the Hoogsteen geometry. R273 side chains interact with the phosphate groups. R248 residues do not interact directly with DNA, but rather with the minor-groove hydration shell described previously (16). Only one dinucleotide pair is shown for type II complexes, as the other pair is related by crystal symmetry. (**E**) In type II complex of R273H/T284R-DNA, the A/T base pairs display the Watson–Crick geometry. T284R side chains interact with the backbone phosphate (also shown in Figure 5C). The R248 side chains form direct interactions with the N3 atoms of the adenine bases. (**F**) In type II complex of R273C/S240R-DNA, the A/T base pairs display the Watson–Crick geometry. S240R side chains interact with the backbone phosphate (also shown in Figure 5D). The R248 side chains form water-mediated interactions with the N3 atoms of the adenine bases.

Interestingly, the interactions of R248 residues with the DNA observed in the rescued complexes are distinctly different from that of the wt complexes (shown in Figure 8A–F). In the wt complexes, R248 residues are relatively distant from the DNA bases and interact occasionally with the DNA backbone in type I structures (13) or via a network of water molecules in type II structures (not shown) (16). In the current complexes, R248 side chains display mostly extended conformations directed toward the minor-groove edges of the DNA bases. In type I complex of R273C/T284R, R248 residues appear occasionally disordered and form water-mediated interactions with the N3 atoms of the two symmetrically disposed adenine bases (Figure 8B and C). In type II complexes of R273H/T284R and R273C/S240R, R248 side chains form direct or water-mediated H-bonds with the adenine N3 atoms, respectively (Figure 8E and F). These findings suggest that the interaction modes of R248 residues at the DNA minor groove are affected by several factors including base-pairing geometry, DNA shape, electrostatic potential and arginine residues interacting with the proximate DNA backbone. R248 is the most frequently mutated amino acid in human cancer (6.6%), predominantly to glutamine and tryptophan (8), and the interaction patterns shown here highlight the role of this amino acid in DNA recognition by p53.

## SUMMARY AND CONCLUSIONS

The crystal structures of the DNA-contact mutants, R273H and R273C, and of the reactivated proteins, demonstrate that p53 inactivation results from the incapacity of the mutated residues to form stabilizing interactions with the DNA backbone, and that p53 rescue can be achieved by alternative protein–DNA interactions formed by the second-site suppressor mutations, T284R and S240R. The high-resolution structural data demonstrate the critical role played by R273 residues of wt p53 in supporting a DNA helix with Hoogsteen base pairs at the center of each half-site, leading to minor-groove narrowing and enhanced electrostatic stabilization of the tetrameric complex. The unique DNA shape is not retained in the rescued complexes, which exhibit the common Watson–Crick base pairs interacting with p53 through direct and water-mediated hydrogen bonds at the minor groove.

As shown here and by the crystal structures of the thermostable core domain incorporating the same DNA-contact mutations (21), the structure and stability of such mutants are highly similar to that of their wt counterparts. An interesting question is whether such mutants can be rescued by small molecules designed to mimic the effect of the DNA-contact suppressor mutations. A potential drug molecule should be located at the protein–DNA interface forming stabilizing interactions with both p53 and its DNA target. This is undoubtedly a greater challenge than the design of drug molecules for the rescue of destabilized p53 mutants as a result of structural mutations (39). However, as reported in recent years, other stabilizing mechanisms by small molecules, yet to be characterized, appear to be effective in the pharmacological reactivation of both DNA-contact and structural mutants of p53 (40).

## ACCESSION NUMBERS

4IBS, 4IJT, 4IBT, 4IBW, 4IBY, 4IBQ, 4IBZ, 4IBU, 4IBV.

## SUPPLEMENTARY DATA

Supplementary Data are available at NAR Online.

Supplementary Data
