# Supplementary material for: Structural studies of p53 inactivation by DNA-contact mutations and its rescue by suppressor mutations via alternative protein–DNA interactions
Source: Nucleic Acids Res. 2013 Jul 17;41(18):8748–59. doi: 10.1093/nar/gkt630 (PMC3794590; doi:10.1093/nar/gkt630)
Supplement: Supplementary Data [file supp_41_18_8748__index.html]

Structural studies of p53 inactivation by DNA-contact mutations and its rescue by suppressor mutations via alternative protein–DNA interactions — Supplementary Data 

# Structural studies of p53 inactivation by DNA-contact mutations and its rescue by suppressor mutations via alternative protein–DNA interactions

## Supplementary Data

files

**Files in this Data Supplement:**

- Supplementary Data - pdf file
