# Supplementary material for: Structural studies of p53 inactivation by DNA-contact mutations and its rescue by suppressor mutations via alternative protein–DNA interactions
Source: Nucleic Acids Res. 2013 Jul 17;41(18):8748–59. doi: 10.1093/nar/gkt630 (PMC3794590; doi:10.1093/nar/gkt630)
Supplement: Supplementary Data [file supp_gkt630_gkt630_suppl_data_July14.pdf]

Structural studies of p53 inactivation by DNA-contact mutations and its rescue by suppressor mutations via alternative protein-DNA interactions

Amir Eldar[1], Haim Rozenberg[1], Yael Diskin-Posner[1], Remo Rohs[2] and Zippora Shakked[1]*
[1]Department of Structural Biology, Weizmann Institute of Science, Rehovot 76100, Israel
[2]Molecular and Computational Biology Program, University of Southern California, Los Angeles, CA 90089, USA

*To whom correspondence should be addressed.
Tel: +972 8 934 2672; Fax: +972 8 934 6278; Email: zippi.shakked@weizmann.ac.il

Supplementary Table S1. Crystallization conditions of R273-related mutants[1].

Crystals	Protein concentration (mg/ml)	Protein/DNA duplex ratio[2]	Reservoir solution		
			Salt (mM)		PEG 3350 (%)
**R273H** (form I)	5		Mg-Formate	120	12
**R273H** (form II)[3]	4	1:1.5	KCl	200	20
**R273H/T284R**	8.1		NaF	200	20
**R273H/T284R-DNA**	5	1:2.4	NH_4F	140	14
**R273H/S240R**	4		Na_2HPO_4	200	20
**R273C**	4		K-Acetate, KF	100,100	20
**R273C/T284R**	6		NH_4-Acetate	120	12
**R273C/T284R-DNA**	3.6	1:2	Li-Acetate	140	14
**R273C/S240R-DNA**	3.5	1:2.4	NH_4F	180	16

[1] In each case, 2μl of protein or protein-DNA solution containing 20 mM sodium citrate pH 6.1, 150 mM NaCl, 10 μM ZnCl2, and 10 mM DTT were mixed with 2μl of the corresponding reservoir solution and equilibrated against 0.5 ml reservoir solution.
[2] DNA sequence cGGGCATGCCCg, consensus sequence underlined.
[3] This crystal form was obtained from a solution of the protein/DNA complex.

Supplementary Figure S1

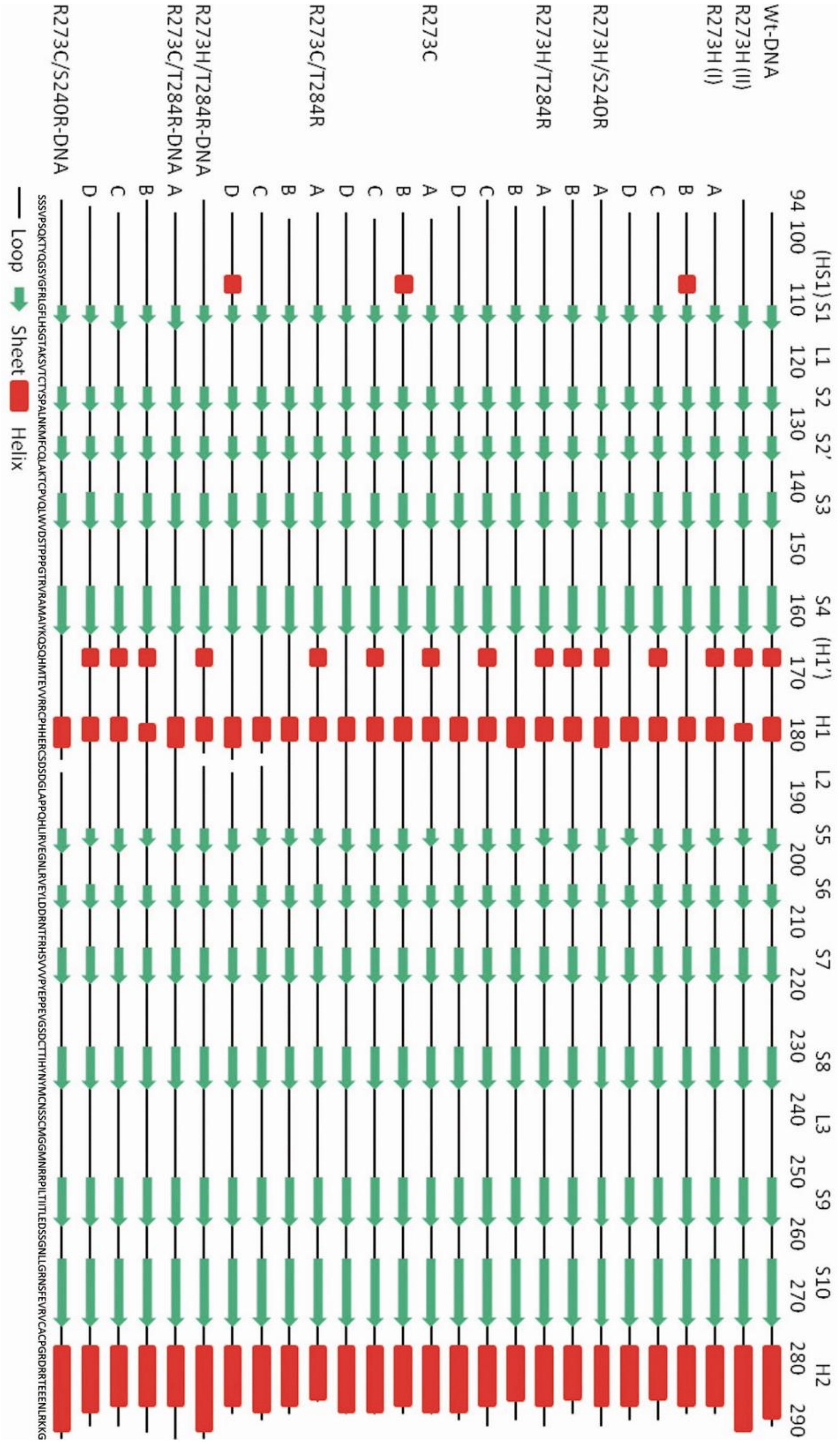

Secondary structures of R273-related mutants. The structures were produced by ProCheck (1). Wild-type p53 structure is based on molecule C of PDB ID 2AC0.

Supplementary Figure S2

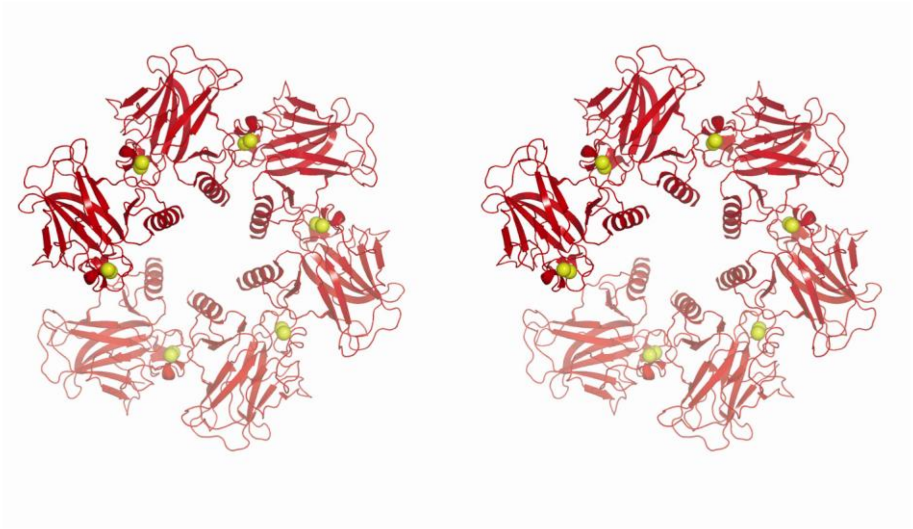

Stereo view of six core-domain molecules of R273H (form II) spiraling around the crystallographic 6_1 axis. The molecules are linked by the second-type Zn atoms (shown as yellow spheres) and intermolecular interactions illustrated in Figure **2B**.

Supplementary Figure S3

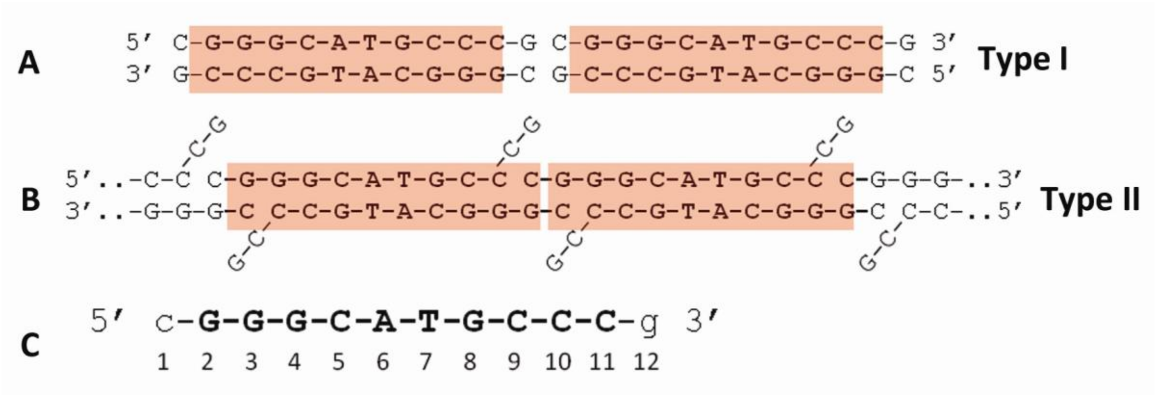

DNA binding sites in type I and type II complexes. Both binding sites are formed by the same DNA dodecamer: 5'-cGGGCATGCCCg-3', incorporating a decameric half-site (underlined).
(**A**) In type I complex, the two half-sites (highlighted by the colored background) are separated by a 2-bp spacer. (**B**) In type II complex, the two half-sites are contiguous. See description in the text.
(**C**) Nucleotide numbering is 1-12 going from the 5' to 3' direction for each strand.

Supplementary Figure S4

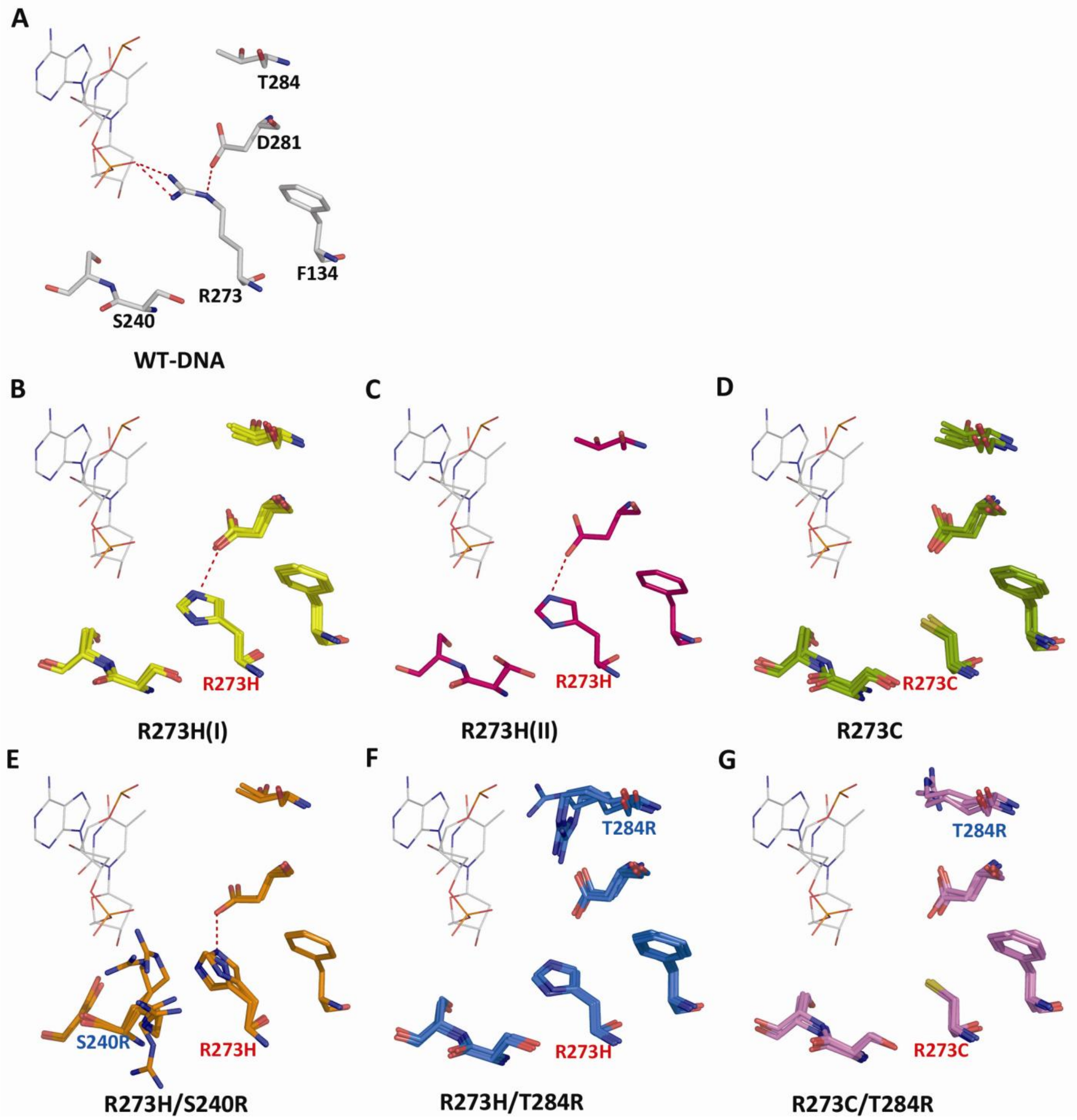

Close-up views of the mutation sites in the oncogenic single mutants and in the rescued double mutants compared to the same region of the wt protein bound to DNA. The DNA part in the views of the mutation sites was modeled by best molecular fitting of each mutant molecule onto the protein part of the wt core domain bound to DNA (PDB ID 2AC0). Except for R273H (form II) with one molecule in the crystallographic asymmetric unit (**C**), all other crystal structures contain two (**E**) or four (**B,D,F,G**) unique molecules superposed on each other. Similarly to **Figure 5** (see text), these views reflects the long distances between the side chains of R273H and R273C to the DNA backbone (close to 4 and 6Å, respectively).

Supplementary Figure S5

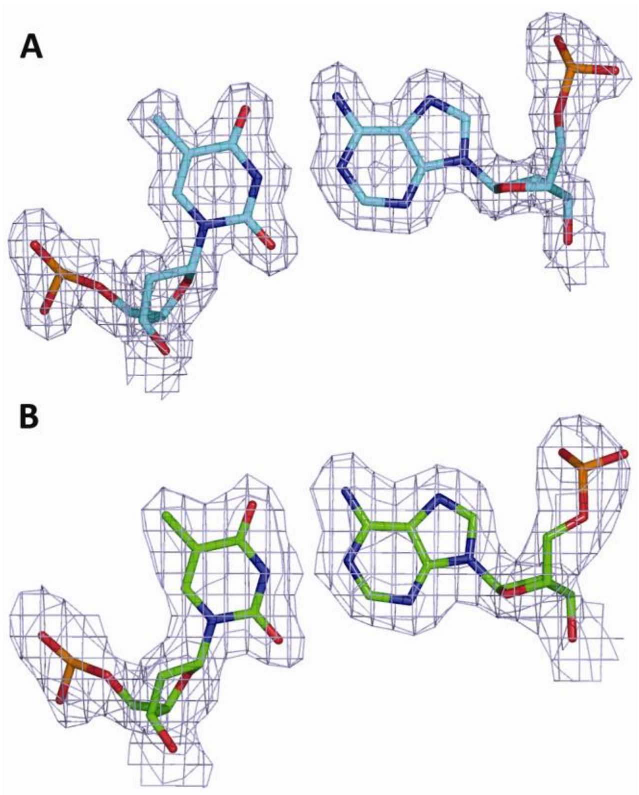

A/T base pairs shown by type-II rescued complexes. R273H/T284R-DNA (**A**) and R273C/S240R-DNA (**B**), display the common Watson-Crick base pairs shown within their respective electron density maps (sigma-A weighted $2F_o$-F_c at 1σ level). This is in contrast to the Hoogsteen base pairs shown by the corresponding complexes of the same DNA targets with p53 core domains where R273 residues remain intact (2). See also discussion in text.

Supplementary References

1. Laskowski R. A., Macarthur M. W., Moss D. S., and Thornton J. M. (1993) ProCheck - a Program to Check the Stereochemical Quality of Protein Structures *J. Appl. Crystallogr.*, **26,** 283-291.

2. Kitayner M., Rozenberg H., Rohs R., Suad O., Rabinovich D., Honig B., and Shakked Z. (2010) Diversity in DNA recognition by p53 revealed by crystal structures with Hoogsteen base pairs *Nat Struct Mol Biol,* **17,** 423-429.
